# The Dynamics of Stress p53-Mdm2 Network Regulated by p300 and HDAC1

**DOI:** 10.1371/journal.pone.0052736

**Published:** 2013-02-20

**Authors:** Akshit Arora, Saurav Gera, Tanuj Maheshwari, Dhwani Raghav, Md. Jahoor Alam, R. K. Brojen Singh, Subhash M. Agarwal

**Affiliations:** 1 Bioinformatics Division, Institute of Cytology and Preventive Oncology, Noida, India; 2 Centre for Interdisciplinary Research in Basic Sciences, Jamia Millia Islamia, New Delhi, India; National Cancer Center, Japan

## Abstract

We construct a stress p53-Mdm2-p300-HDAC1 regulatory network that is activated and stabilised by two regulatory proteins, p300 and HDAC1. Different activation levels of 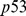 observed due to these regulators during stress condition have been investigated using a deterministic as well as a stochastic approach to understand how the cell responds during stress conditions. We found that these regulators help in adjusting p53 to different conditions as identified by various oscillatory states, namely fixed point oscillations, damped oscillations and sustain oscillations. On assessing the impact of p300 on p53-Mdm2 network we identified three states: first stabilised or normal condition where the impact of p300 is negligible, second an interim region where p53 is activated due to interaction between p53 and p300, and finally the third regime where excess of p300 leads to cell stress condition. Similarly evaluation of HDAC1 on our model led to identification of the above three distinct states. Also we observe that noise in stochastic cellular system helps to reach each oscillatory state quicker than those in deterministic case. The constructed model validated different experimental findings qualitatively.

## Introduction

The p53 is a 20-Kb tumor suppressor gene located on the small arm of human chromosome 17 that acts as a hub for a network of signalling pathways essential for cell growth regulation and apoptosis. It comprises of 393 amino acids and is divided into several structural and functional domains: the transactivation domain (TAD; residues 1–40), the proline-rich domain (PRD; residues 61–94), the DNA-binding domain (DBD; residues 100–300), the tetramerization domain (4D; residues 324–355) and the C-terminal regulatory domain (CTD; residues 360–393) [1]. Over the recent years many names have been accredited to p53 viz. Guardian of the Genome [2]; Death Star [3] and Cellular Gatekeper [4] and is regulated by a number of cellular proteins [5]. It is well established that p53 is accountable for preventing improper cell proliferation and maintaining genome integration following genotoxic stress. In normal proliferating cells, p53 is kept in low concentrations and exists mainly in an inactive latent form with a short half-life of 15–30 minutes [6]. This is due to interaction between p53 and Mdm2 the predominant negative regulator of p53. However, cellular insults activates p53 and its level increases rapidly. The activation of p53 is a result of several posttranslational modifications including phosphorylation, acetylation, sumoylation and neddylation [7]. Phosphorylation of Ser-15 and 37 at the amino terminus of p53 prevents Mdm2 binding, thus stabilizing p53. Also phosphorylation at Ser-15 increases p53 affinity for p300, thus promoting acetylation of p53 carboxy terminal by p300 [8]. Further the p53 in-turn activates the p53-targeted genes including those involved in cell cycle arrest and DNA repair, as well as apoptosis and senescence related genes. The activation of the p53-targeted genes leads to cell cycle arrest that forces cell to choose either to repair the DNA damage to restore its normal function or cell death (apoptosis). Further, it has been observed that p53 acetylation is a reversible process and for it Mdm2 recruits HDAC1 (a histone deacetylase) to form a Mdm2-HDAC1 complex which deacetylates p53. Interestingly, it was also shown that p300 can form a complex with Mdm2 in vitro and in vivo [9], [10] and this complex (Mdm2-p300) facilitate Mdm2 mediated p53 degradation. Moreover, it has also been reported that Mdm2-p53-p300 complex exists that is also thought to promote ubiquitylation and degradation of p53 [11]. Thus p300 plays dual role and exerts two opposite effects on p53 in cells i.e., it can either interact with Mdm2 promoting Mdm2-mediated ubiquitylation and degradation of p53 [9] or acetylate and stabilize p53. This remains puzzling.

There have been different mathematical techniques to study cellular and sub-cellular processes such as deterministic and stochastic models [12], [13]. Stochastic model provide detail picture of molecular interaction in the microscopic systems (small systems with small number of molecules accomodated in each system) that leads the system dynamics as noise-driven process [13], [14]. The model further highlights the important role of noise in the system dynamics, for example detection and amplification of weak noise, the phenomenon known as stochastic resonance [15], [16], lifting of cellular expression at different distinct expression state [17] and noise in gene expression can drive stochastic switching among such states [18], [19], noise induced stochastic phenotypic switching to different new level in living cells [20] etc. However, deterministic model provides qualitative picture of the cellular or sub-cellular processes.

The aim of the present study is (i) to understand some of the basic issues of p53 autoregulation induced by regulators p300 and HDAC1, (ii) to elucidate the functional relationship of p300 and HDAC1 in regulating p53 function, (iii) how do these regulators lifts the normal p53-Mdm2 network to different stress states and (iv) what could be the role of noise in such situations.

## Materials and Methods

### Stress 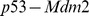 model regulated by 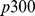 and 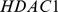


In normal proliferating cells, p53 is usually maintained at low levels due to p53 and Mdm2 protein feedback mechanism [21]. In unstressed condition the p53 binds to the regulatory region of Mdm2 gene and stimulates its transcription into messenger RNA (mRNA) with a transcription rate constant 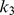, followed by translation into Mdm2 protein with a rate constant 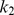 [22]. The degradation of Mdm2-mRNA, Mdm2 and genesis of p53 occurs with basal rate of 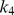, 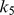 and 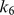 respectively. The Mdm2 protein then interacts physically with p53 to form Mdm2-p53 complex with the rate of 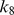. Mdm2 functions as an E3 ubiquitin ligase and brings about ubiquitylation of multiple lysine residues (K370, K372, K373, K381, K382 and K386) [23] present in the C-terminal domain of p53 [11]. The ubiquitylation marks p53 for degradation via the 26S proteasome, with rate 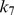. The Mdm2-p53 complex can also dissociate to Mdm2 and p53 with rate constant 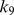. Mdm2 and p300 have been shown to interact with rate constant 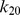 to form Mdm2-p300 complex, which facilitates p53 polyubiquitination and degradation at rate constant of 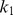 [9], [24]. Although there is no direct evidence reported to the best of author's knowledge in the literature for the degradation of Mdm2-p300 complex, however it has been shown that the p19ARF-binding domain of Mdm2 overlaps with its p300-binding domain suggesting that p19ARF could interfere with the Mdm2/p300 interaction [9]. Therefore, we can assume it is possible that Mdm2-p300 complex can be broken so as to interact with other proteins. Thus in normal unstressed cell, p53 is maintained at low level in an active state with short half-life of 15–30 minutes by Mdm2 and the cells are able to proliferate.

**Figure 1:**
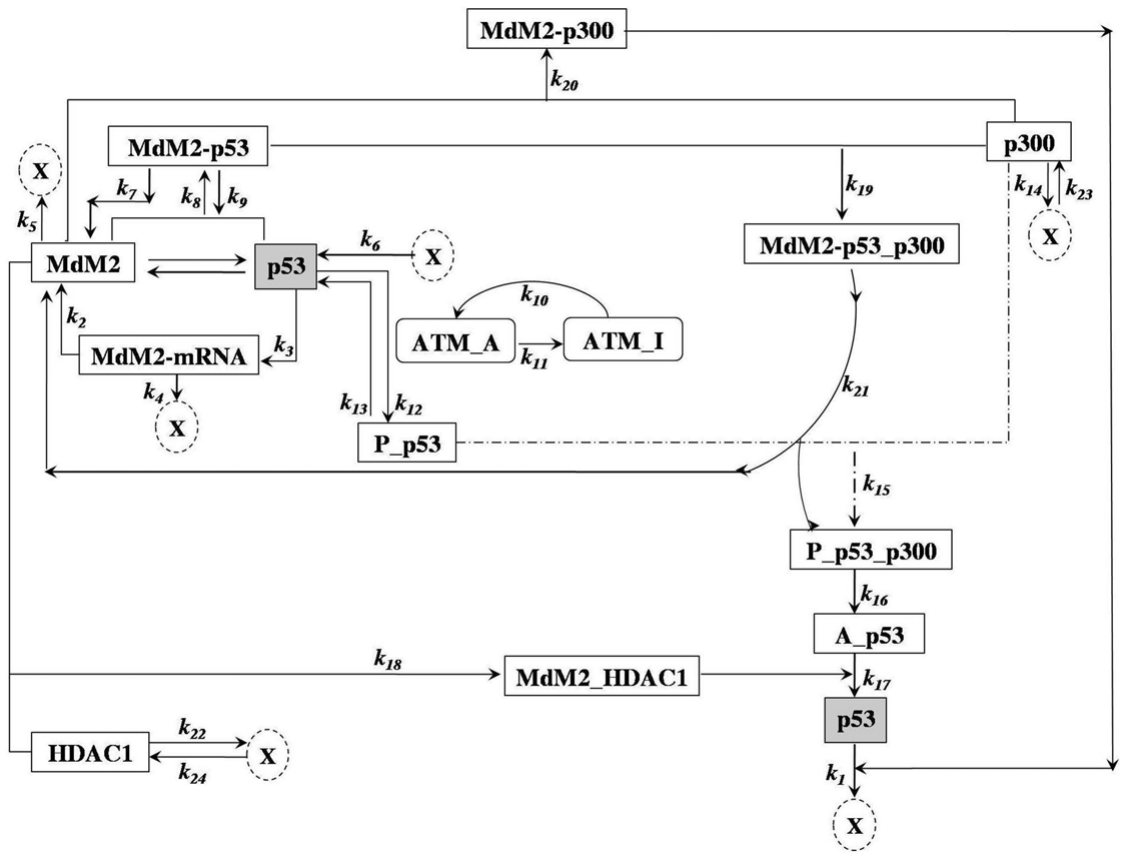
Biochemical network model of stress p53-Mdm2-p300-HDAC1. The schematic diagram of stress p53-Mdm2-p300-HDAC1 model.

However, under stressed conditions the p53 is stabilized through various post translational modifications which lead to increase its level. Of the various mechanisms, phosphorylation of 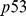 is the most well studied and it is reported that multiple kinases phosphorylate various residues which increase the level of 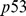 protein. One of these protein kinases is 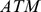 which upon activation by DNA damage phosphorylates 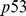 with a rate 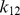 at serine 15 [25] which is critical for 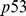 activation and stabilization. Strikingly, the phosphorylation of serine 15 mediated by 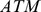 acts as a nucleation event that promotes subsequent sequential modification of many residues. To achieve this, interconversion of inactivated and activated 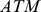 takes place, with rate constants 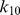 and 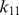 respectively. The 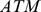-initiated phosphorylation reduces the affinity of 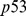 for 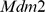 while increases interactions with HATs like 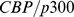 [8], [26]. Consequently, dephosphorylation of 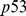 with a rate 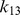 also takes place to counter this phosphorylation. It has been demonstrated that 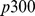 protein is a co-activator of 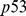 which potentiates its transcriptional activity as well as biological function in vivo [27]. However, it has also been shown that formation of 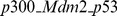 ternary complex leads to suppressing 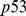 acetylation and activation [28]. The transcription activation domain (TAD) of 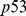 binds tightly to 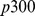 with formation rate constant 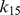. The 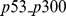 complex hence formed, causes 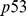 acetylation with rate constant 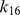 at multiple lysine residues (K370, K372, K373, K381, K382) of its C-terminal regulatory domain [27], [29]. The lysine residues (K370, K372, K373, K381, and K382) are the common sites for both acetylation and ubiquitination [30], [31]. Thus their acetylation causes the inhibition of ubiquitination resulting 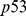 protein stability which is evident from the observation that acetylated 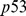 has half-life of greater than two hours [32]. Simultaneously, formation and degradation of 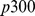 occurs with rate constants 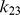 and 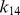 respectively. 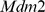, 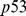 and 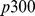 have also been demonstrated to exist in a ternary complex (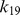) which is incapable of acetylating 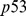 [28]. In the complex, TAD1 domain of 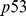 interacts with 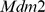 while TAD2 interacts with 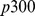 [11]. As mentioned earlier, phosphorylation increases the affinity of 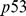 towards 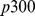 while decreasing its affinity towards 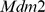. After phosphorylation, the ternary complex dissociates, with rate constant 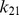 into 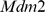 and 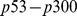 complex, in which both TAD1 and TAD2 of 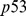 interact with 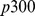 [11]. p300 can then acetylate and stabilize 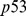. Stabilized 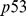 functions as a tumor suppressor and induces high levels of 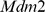, which in turn promotes 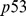 degradation by recruiting a 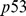 deacetylase, 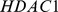 with rate constant 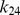. 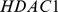 binds 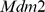 in a 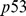 dependent manner with binding rate constant 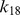 and deacetylates 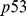 with rate constant 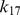 at all known acetylated lysines in vivo [33]. Moreover, analysis has indicated the presence of MDM2, SMAR1 and HDAC1 complex under conditions of inhibited translation only 12 h post damage rescue while there is lack of complex formation 24 h post damage rescue, thereby suggesting degradation of the Mdm2-HDAC1complex [34]. HDAC1 is generated and degraded in cells with rate constants 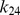 and 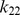 respectively. The unmodified lysine residues can then serve as the substrates for 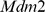-mediated ubiquitylation resulting in 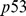 degradation and thus completing the feedback loop. The molecular species involved in the biochemical network are listed in Table 1 and the chemical reaction channels in the network are shown in Table 2. The schematic picture of the stress 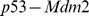 autoregulatory biochemical reaction network model via 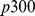 and 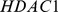 based on the experimental evidences and reports mentioned above is shown in Fig. 1.

**Table 1:** List of molecular species.

S.No.	Species Name	Description	Notation
1.	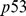	Unbounded 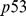 protein	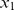
2.	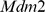	Unbounded 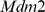 protein	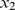
3.	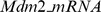	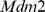 messenger 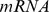	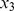
4.	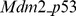	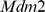 with 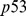 complex	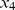
5.	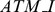	Inactivated 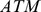 protein	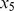
6.	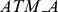	Activated 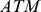 protein	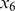
7.	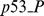	Phosphorylated 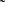 protein	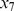
8.	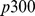	Unbounded 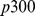 protein	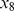
9.	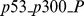	Phosphorylated 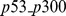 complex	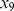
10.	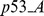	Acetylated 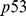 protein (capped p53)	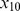
11.	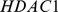	Unbounded 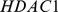 protein	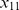
12.	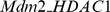	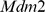 and 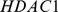 complex	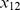
13.	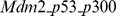	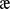 , 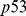 and 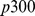 complex	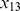
14.	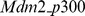	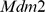 and 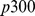 complex	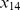

**Table 2:** List of chemical reaction, propensity function and their rate constant.

S.No	Reaction	Name of the process	Kinetic law	Rate constant	References
1	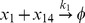	p53 degradation	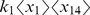	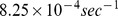	[9], [24].
2	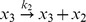	Mdm2 creation	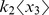	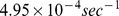	[22].
3	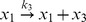	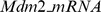 creation	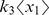	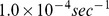	[22].
4	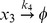	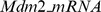 degradation	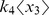	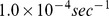	[22].
5	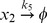	Mdm2 degradation	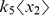	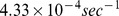	[22].
6	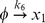	p53 synthesis	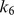	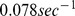	[22].
7	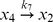	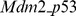 degradation	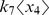	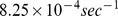	[11], [23].
8	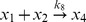	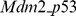 synthesis	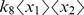	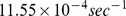	[22].
9	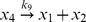	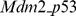 dissociation	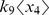	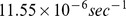	[22].
10	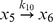	ATM activation	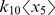	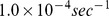	[12], [23].
11	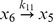	ATM deactivation	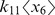	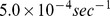	[12], [23].
12	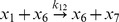	Phosphorylation of p53	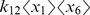	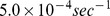	[23].
13	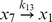	Dephosphorylation of p53	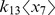	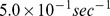	[12], [23].
14	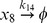	p300 degradation	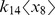	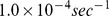	[30], [31].
15	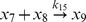	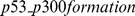	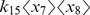	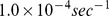	[28].
16	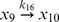	Acetylation of p53	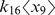	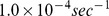	[27], [29].
17	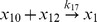	Deacetylation of p53	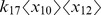	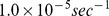	[29].
18	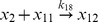	Creation of 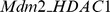	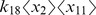	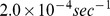	[29].
19	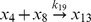	Creation of 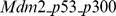	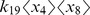	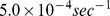	[28].
20	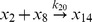	Formation of 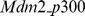	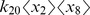	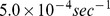	[9], [22].
21	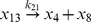	Dissociation of 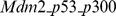	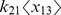	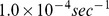	[11], [28].
22	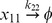	Degradation of HDAC1	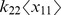	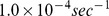	[29].
23	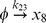	p300 synthesis	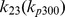	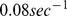	[30], [31].
24	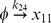	HDAC1 synthesis	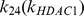	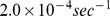	[29].

### Stochastic description of biochemical reaction network

We now consider a configurational state 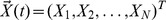 of the system of size 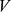 at any instant of time 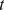 defined by 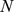 molecular species undergoing 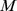 elementary reactions. The change in configurational state during any interval of time 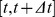 is due to random interaction of the participating molecules that leads to decay and creation of specific molecular species in state vector 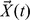 during the time interval [13], [14], [35]. Therefore the trajectory of this state vector 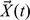 as a function of time in the configurational space follows Markov process [13], [14] and the dynamics of this vector becomes noise-induced stochastic process [13]. If we define 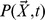 as the configurational probability of obtaining the state 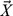 at time 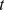, then the time evolution of 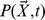 will obey Master equation [13], [14], [36]. Even though the Master equation for complex system could be very difficult to solve analytically, different algorithms have been devised to solve the system dynamics numerically depending on the nature of the system. For example, stochastic simulation algorithm (Gillespie algorithm) for reaction system without considering time delay [13], stochastic simulation algorithm for time delay reaction system [37], [38], 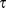-leap algorithm which is approximated algorithm of stochastic simulation algorithm for very complex reaction network [39], hybrid algorithm for reaction networks consisting of both slow and fast reactions [40] etc.

The Master equation for the stochastic system can be approximated to simpler Chemical Langevin equations (CLE) based on two important realistic approximations applied on the the system [41]. This can be done by defining a function 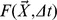 which is the number of a particular reaction fired during the time interval 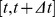 with 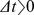 and applying the two approximations: first applying 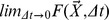 which leads to the prophensity functions (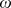) of the reactions fired remain constant during the time interval, and secondly applying 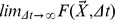 condition which gives rise 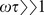 [41]. These two conditions are true for large population size of each variables in state vector 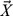 which is valid for natural systems. These two conditions allow the function 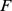 to approximate to Poisson distribution function and then to Normal distribution function with same mean and standard deviation. If molecular concentration is defined by 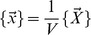 and linearize Normal distribution function, the Master equation leads to the following CLE of the vector 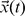,
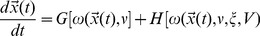
(1)where, 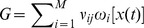 is the macroscopic contribution term and 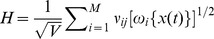 is the stochastic contribution term to the dynamics. 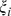  =  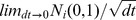 is uncorrelated, statistically independent random noise parameters which satisfy 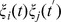  =  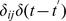. {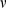} is the stoichiometric matrix of the reactions in the network.

The classical deterministic equations can be obtained from the CLE equation (1) at thermodynamics limit [41] i.e. at 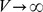, 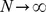 but 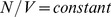. This leads to 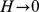 and the equation (1) becomes noise free deterministic equation,
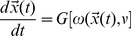
(2)


The same equation (2) can also be be retrieved from the biochemical reaction network by translating them into a set of differential equations based on standard principles of Mass-action law of biochemical reaction kinetics.

The stress 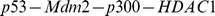 model network we study is defined by 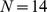 (14 molecular species) and 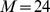 (24 reaction channels). The molecular species, possible reactions, kinetic laws and the rate constants in this model are listed in Table 1 and Table 2 respectively. The state vector at any instant of time 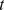 is given by, 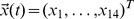, where the variables in the vector are various proteins and their complexes which are listed in Table 1. The classical deterministic equations constructed from these reaction network are given by,
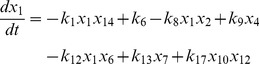
(3)

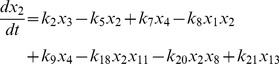
(4)

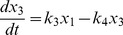
(5)

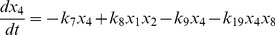
(6)

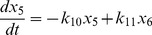
(7)

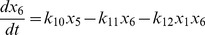
(8)

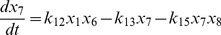
(9)

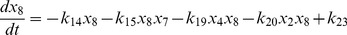
(10)

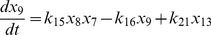
(11)

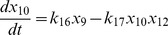
(12)

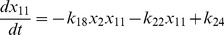
(13)

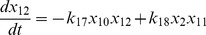
(14)

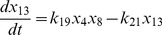
(15)

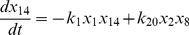
(16)where, 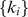 and 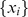, 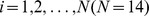 represent the sets of rate constants of the reactions listed in Table 2 and concentrations of the molecular populations listed in Table 1.

Following the same procedure as we have discussed above, we reach the following CLE for the network shown in Fig. 1, Table 1 and Table 2.



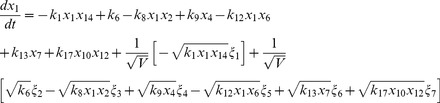
(17)




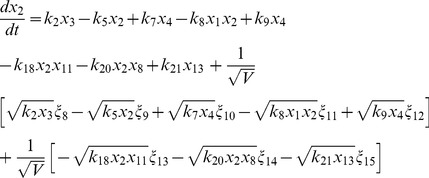
(18)

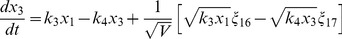
(19)

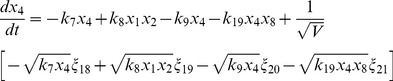
(20)

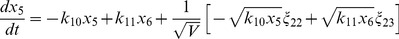
(21)

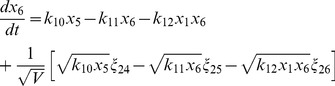
(22)

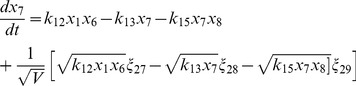
(23)

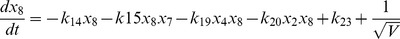


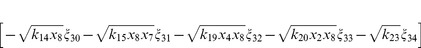
(24)

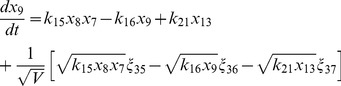
(25)

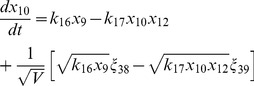
(26)

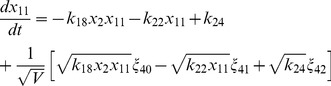
(27)

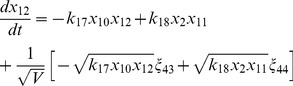
(28)

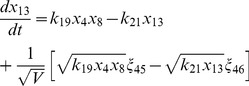
(29)

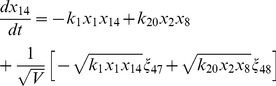
(30)where, 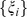 are random number which satisfy 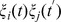  =  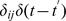, and 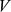 is the system's size.

The CLE (3)-(17) and differential equations (18)-(32) can be solved numerically using standard algorithm of 4th order Runge-Kutta method of numerical integration [42].

## Results and Discussion

Several researchers have studied the oscillations of 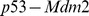 network in detail [22], [43]–[46], however to the best of our knowledge this study is the first one that uses systems biology approach for understanding the complex role of p300 and HDAC1 on p53. We numerically solved the set of deterministic differential equations (1)–(14), and stochastic CLE (15)–(29) by using standard algorithm of 4th order Runge-Kutta method of numerical integration [42]. We thus study the impact of p300 and HDAC1 on p53 activation and stabilization to understand the fate of the cell.

### Impact of 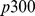 on 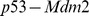 activation

**Figure 2:**
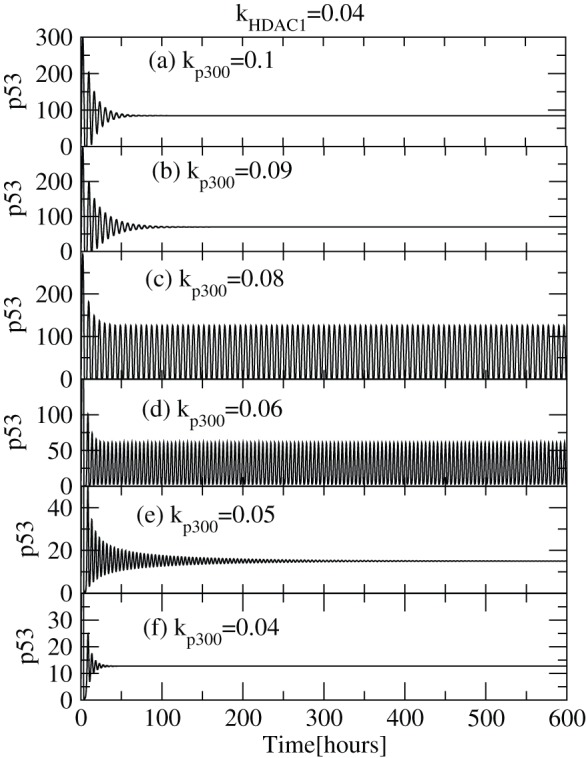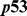 dynamics for various 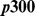 levels. The plots of 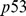 concentration levels as a function of time in hours for various 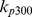 values: (a) 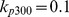, (b) 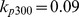, (c) 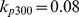, (d) 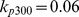, (e) 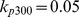 and (f) 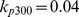 respectively at constant value of 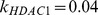.

We first present the deterministic results on p53-Mdm2 regulatory network on exposure to different concentrations of 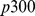 i.e. at different rate constants, 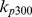 (Fig. 2). For small values of 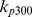 ( = 0.04) (lower 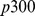 concentration), 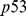 is first activated for some time (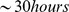) and then resumes its normal condition indicated by its constant level (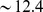) which is the level of stabilization. The range of activation is increased as 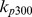 increases (increase of 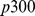 concentration) as well as there is rise in the level of stabilization. However, when 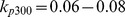, 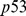 maintains sustain oscillations which leads to increasing level of activation as a consequence. With further increment of 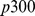 concentration level, 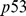 dynamics that was at sustain oscillations switched to damped oscillations and subsequently p53 concentration is stabilized at a constant level. This activity suggests that the capping of the c-terminal of 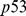 is higher and there is no decrement in the 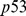 levels as a result of which 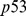 is stabilized. The results obtained are consistent with the experimental observations which indicates that acetylation of p53 is responsible for its activation [27], [31] and stabilization [29], [32]. If we further increase the value of 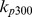, 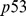 activation decreases maintaining 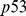 stability but at higher values (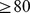). Hence we identify two conditions where p53 is stabilized, one at lower values (nearly normal cell condition) and the other at larger values (cell death condition) of 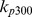 and in between 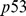 is activated.

**Figure 3:**
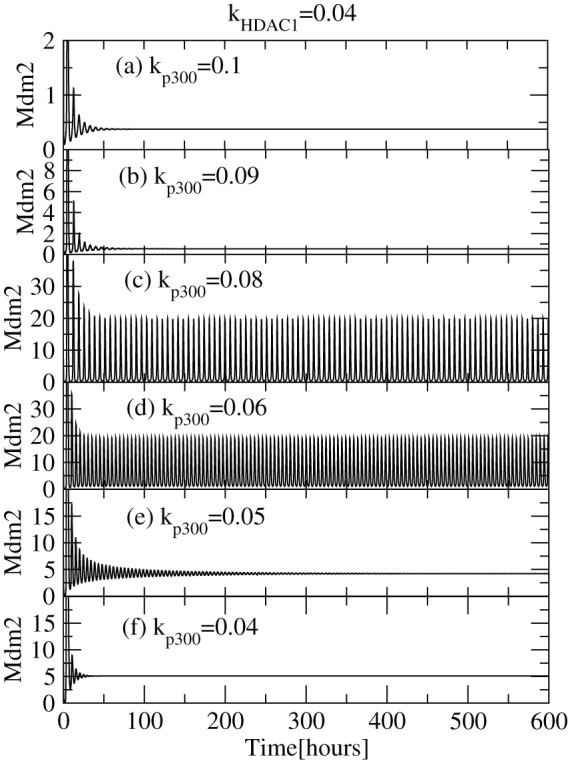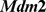 dynamics for various 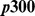 levels. The plots of 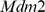 concentration levels as a function of time in hours for various 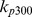 values: (a) 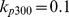, (b) 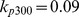, (c) 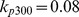, (d) 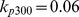, (e) 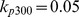 and (f) 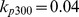 respectively at constant value of 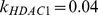.

**Figure 4:**
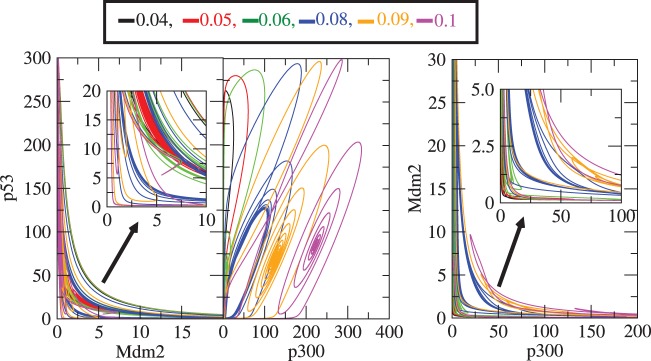
Two-dimensional recurrence plots of 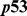 and 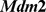. Recurrence plots between (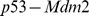), (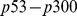) and (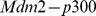) for different values of rate constants 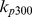, i.e. 0.04, 0.05, 0.06, 0.08, 0.09 and 0.1 respectively.

Similarly, 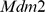 dynamics as a function of time for different values of 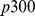 concentration levels is shown (Fig. 3) that demonstrates counter behaviour as expected. The two dimensional recurrence plots of (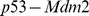), (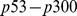) and (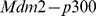) are presented in Fig. 4 which provides clear and qualitative picture of the above facts. The emergence of sustain/limit-cycle oscillation (activated 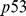 level) from fix point oscillation (stabilized 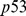 level), and then from sustain oscillation to again fix point oscillation is observed as one increase the concentration of 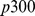.

### Impact of 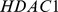 on 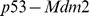 network

**Figure 5:**
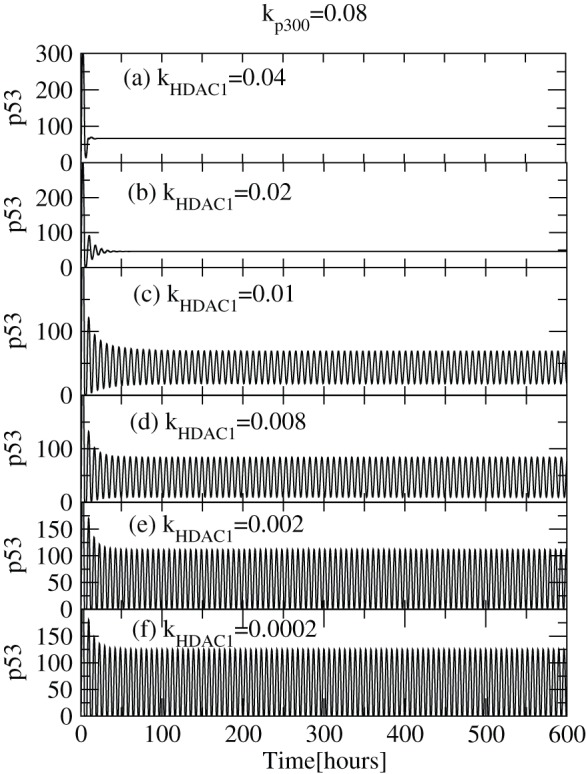
Activation of 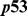 via variation of 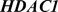 level. Plots of 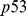 concentration levels as a function of time in hours for various 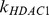 values 0.0002, 0.002, 0.008, 0.01, 0.02 and 0.04 respectively (at constant value of 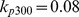), showing activation and stabilization of 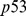.

Several studies suggest that 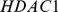 is involved in the deacetylation of p53 which has a potent impact on 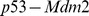 regulatory dynamics [29], [31], [47], [48]. It has been found that 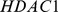 makes complex protein, 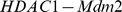 which deacetylates and then ubiquitinates the acetylated 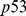. Because of this process of interaction of 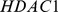 with 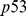, both 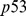 and 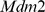 levels get stabilized. In our numerical simulation, we kept 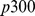 concentration level fixed by keeping 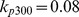 throughout the simulation and allow 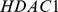 concentration to vary by changing 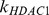 value. The results are shown in Fig. 5 (a)–(f). In these plots we observe that at lower concentration of 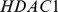 (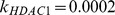), the 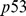 activation is large due to pre-existing 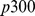, as indicated by the sustained oscillation (Fig. 5 (f)). This activity suggests that there is regular decay and creation of 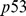, due to the presence of high levels of 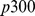 and hence the impact of 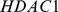 concentration level is not very significant. As the 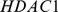 concentration increases (increasing 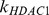 value), there is regular and competitive effect between 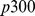 and 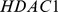 for 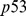 that decreases 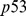 activation as indicated by decrease in 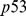 concentration level (Fig. 5 (c)–(e)). Further, if we increase the concentration of 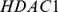, the 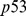 first activates for short period of time and then remains constant at same value (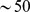) indicating 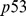 stabilization. This transition from 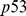 activation to stabilization is indicated by the transition from sustained oscillation to fixed point oscillations indicated in Fig. 5 (a) and (b). We observe this behaviour at 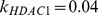, where the activity of 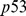 is low, stable and very much controlled.

**Figure 6:**
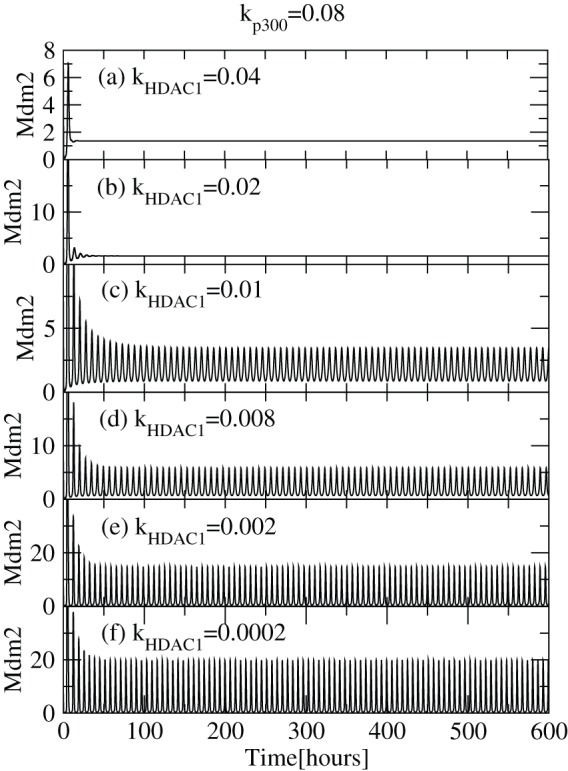
Activation of 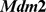 via variation of 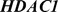 level. Plots of 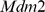 concentration levels as a function of time in hours for various 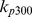 values 0.0002, 0.002, 0.008, 0.01, 0.02 and 0.04 respectively (at constant value of 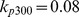), showing activation and stabilization of 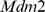.

Similarly, we present the simulation results of 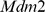 as a function of time for different 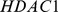 levels (Fig. 6 (a)–(f)). We observe similar behaviour of 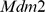 as 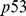 which shows a transition from sustain oscillation to fix point oscillation as one increase the 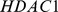 concentration level. These results indicate that 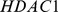 stabilizes 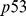 as well as 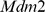 concentration levels.

**Figure 7:**
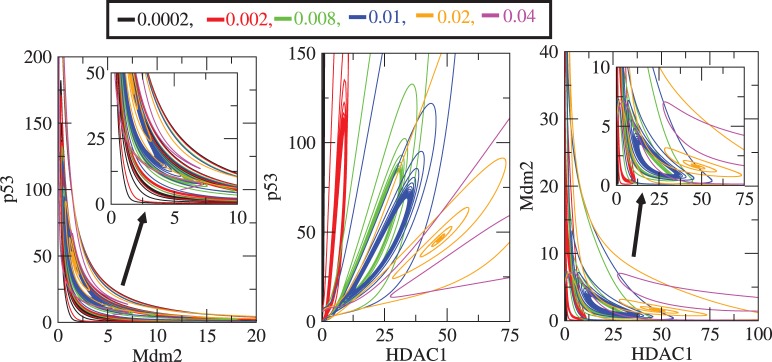
Recurrence plots of  and 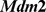 activated by 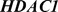. The two dimensional plots of the pairs (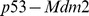), (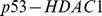) and (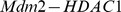) for different values of rate constants 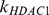, i.e. 0.0002, 0.002, 0.08, 0.01, 0.02 and 0.04 respectively.

We also present the two dimensional recurrence plots of the (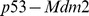), (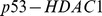) and (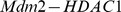) for demonstrating these facts (Fig. 7). The clear indication of transition from sustain/limit cycle oscillation to fix point oscillation as 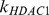 is increased, is shown in the plots indicating transition from activation of 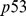 and 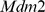 to stabilized state.

### Stability analysis of 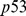 and 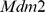


**Figure 8:**
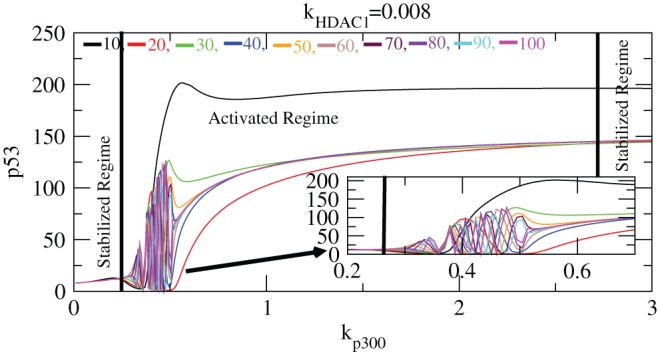
Stability curve induced by 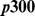. Plots of 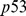 concentration level as a function of 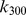 for different values of exposure times i.e. 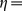
10-100 (at constant value of 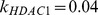). The inset is the enlarged portion of the activated regime. In the curve stabilized and activated regimes are demarcated.

We then checked how concentration level of 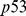 varies as a function of 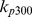 (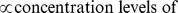 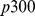). This is done by defining a parameter called expose time (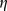) which can be stated as the amount of time the system is exposed to a particular concentration level of 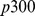 or 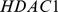. The calculation of 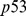 or 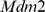 concentration level induced by the exposition of the system to 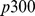 or 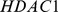 is done by obtaining its level just after the expose time (time slice calculation). Fig. 8 shows variation of 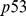 concentration levels as a function of 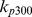 for different expose times of 10–100 hours for a fixed value of 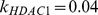. The plots clearly show the activated and stabilized regimes. The activated regime is where the 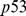 levels fluctuate as a function of 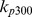 (induced by 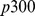 levels). In the plots, 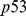 level starts activation from 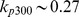 because of the interaction among 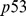, 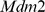 and 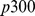 with small level of 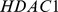 giving rise to fluctuation in 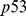 level. This could be due to acetylation and deacetylation which leads to capping (which prohibits 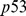 to decay) and uncapping (which leads to 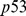 decay) of 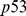 due to 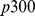. This 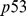 level fluctuation persists till 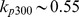 and then increases its level without fluctuation till 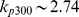 indicating only the capping of 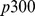, then its level remain constant. Interestingly the range of activation of 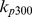 in 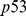 for all expose times remain the same in [0.27–2.74].

The stabilized regimes are where 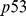 level is not affected by the variation in 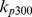 (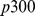 level variation). Initially, within the range of 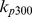 [0–0.27], the 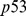 level is not much affected indicating that the cell resumes its normal condition maintaining its minimum level (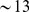) which we call first stabilization regime. However, in the second stabilization regime [2.74–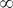], 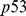 level remains constant at much higher value (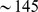) indicating the capping of 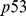 is maximum utilizing 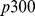 which prohibits decay. This case may be the condition where death of the cell could happen due to uncontrolled 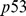 growth due to excess 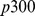.

**Figure 9:**
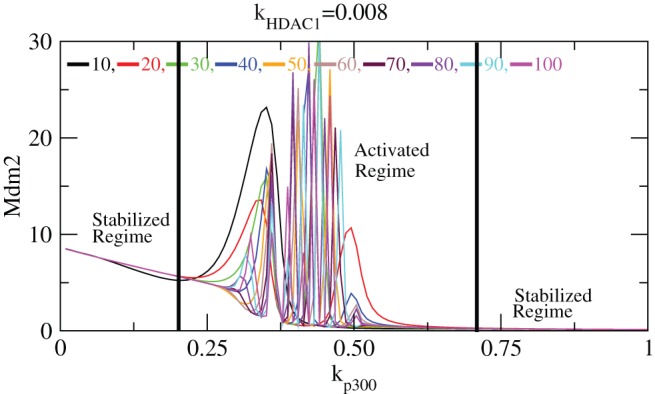
Stability curve induced by 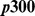. Plots of 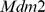 concentration level as a function of 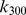 for different values of exposure times i.e. 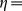
10–100 (at constant value of 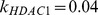). In the curve stabilized and activated regimes are demarcated.

The activation and stabilization of 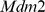 induced by 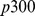 is shown in Fig. 9. Since 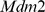 is counter part of 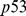 which is activated by 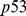, similar results are obtained as in the case of 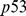. The first stabilization regime is within [0–0.23] values of 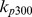, followed by activation regime [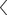
0.23–0.7] and finally second stabilization regime [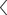
0.7–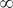]. The increased level of 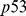 in the second stabilization regime are capped 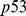 level which are prohibited from decay and taking part in any other reactions and therefore is not able to activate 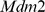 level. Hence its level reduces to minimum as soon as the second stabilization regime is reached.

**Figure 10:**
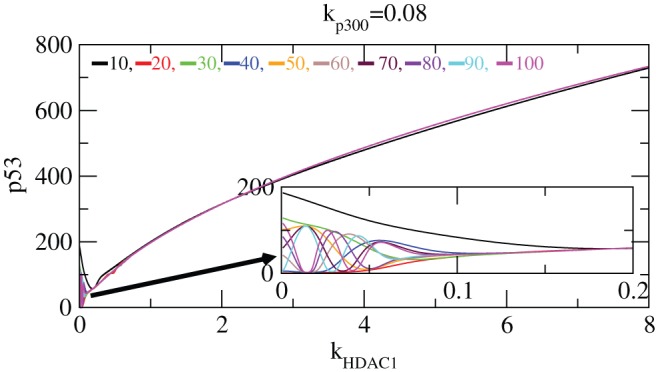
Stability curve induced by 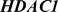. The variation of 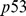 concentration level versus 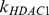 for different exposure times 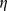 = 10–100, keeping 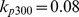 fixed. The inset is the enlarged portion of the actively activated regime.

**Figure 11:**
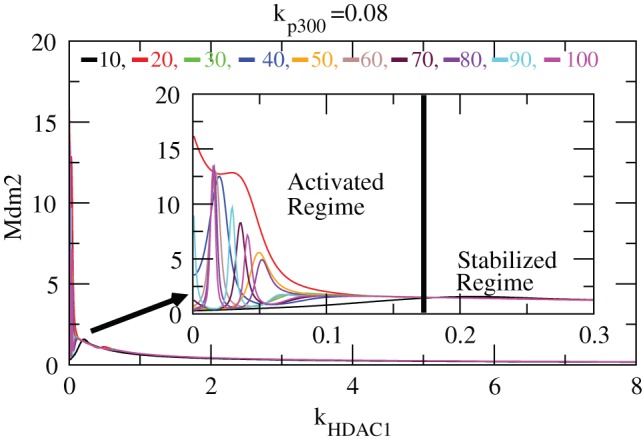
Stability curve induced by 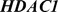. The variation of 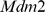 concentration level versus 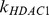 for different exposure times 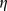 = 10–100, keeping 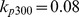 fixed. The inset is the enlarged portion of the activated and stabilized regimes.

Next we study the impact of 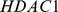 on 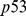 stabilization in our system. This is done by keeping the value of 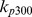 fixed at 0.08 and simulating the level of 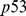 as a function of 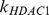 for different exposure times 10–100 hours (Fig. 10). From the plots one can see the activation of 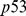 at low 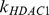 values due to 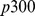 impact but not due to 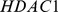 contribution. As 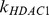 value increases, the 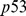 level starts decreasing due the deacetylation of 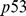 which allow it to degrade and take part in reactions. The activation of 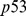 with fluctuation persists till 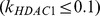. After 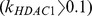, 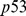 level remains constant for a short period of time and then its level starts increasing without fluctuation. This behaviour indicates that 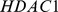 has suppressing impact on 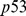 activation. This pattern is same for all exposure times as is shown in the plots (Fig. 10). The same pattern is found for 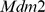 also which in fact is the counterpart of 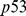. The activated and stabilized regimes are shown in the Fig. 11.

**Figure 12:**
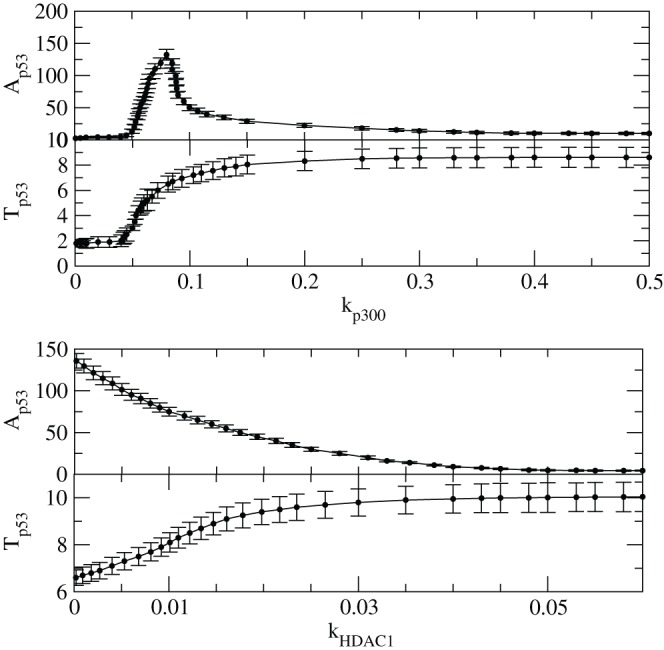
The variation of 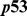 amplitude and time period induced by 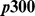. (a) Plots of 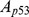 and 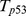 as a function of 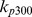 (upper two panels) which capture the stabilized and activated regimes. (b) Plots of 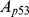 and 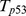 as a function of 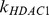 (lower two panels) which capture the stabilized and activated regimes.

**Figure 13:**
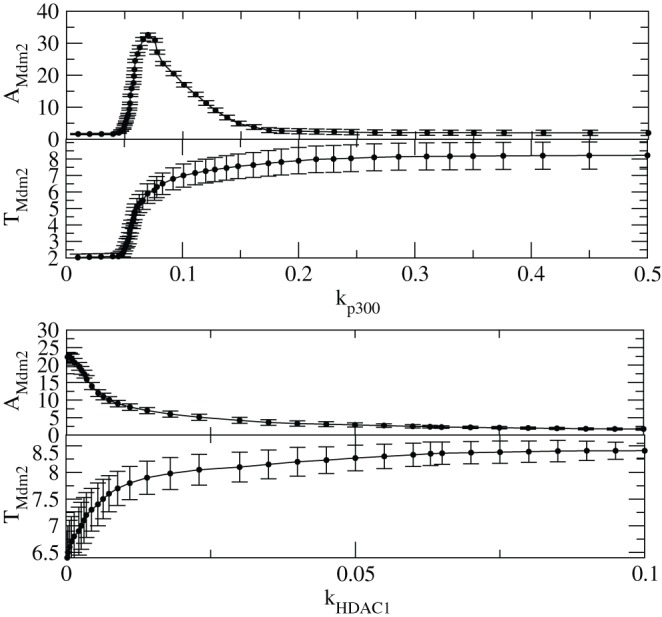
The variation of 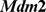 amplitude and time period induced by 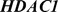. (a) Plots of 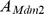 and 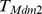 as a function of 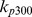 (upper two panels) which capture the stabilized and activated regimes. (b) Plots of 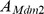 and 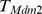 as a function of 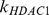 (lower two panels) which capture the stabilized and activated regimes.

We then present the results of amplitudes of 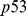, (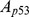) and time period, (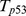) as a function of 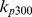 and 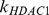 to understand the how 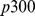 and 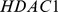 influence the amplitude and time period of 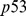 oscillations (Fig. 12). The calculation of 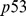 amplitude is done as in the following. For sustain oscillation we took time range of [100–200] hours in our calculation and then calculated the average of it. Then we take 50 such time series for different initial conditions and determine the average of p53 amplitude again (Fig. 12 and 13). The points in the plots are average points with error bars. For damped oscillations, we take the available number of oscillations and calculated the average of those oscillations which is found to be equivalent to the distance between x-axis and line which shows no oscillation (stable line) approximately. Similarly, for stabilized regime we determine distance between x-axis and stable line for each values of 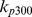 or 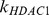 and average over 50 time series. Initially, 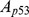 remains constant at lowest value for small values [0–0.05] of 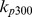, then it monotonically increases and decrease in the interval [0.05–0.3] and finally its value remains constant. This in fact is the consequence of first stability (normal condition) where the impact of 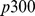 is negligible, then activation of 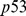 due to interaction of 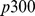 with 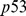 and other proteins and then stabilization of 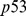. These three regimes can also be seen in the case of 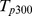 versus 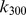 plot.

However, in the case of 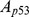 and 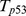 induced by 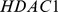, the first stability condition is not observed because the cell is already activated with a constant 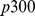 level i.e. at constant 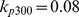. In this case 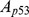 level decreases as 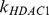 increases till 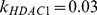 and the remains constant. However, 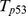 increases till 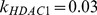 and then stabilized.

Similarly we calculated 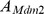 and 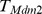 as a function of 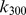 and 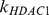 respectively and the results are shown in Fig. 12. For both the parameters similar behaviour was obtained as in the case of 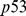.

### Deterministic steady state solutions: impact of 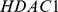 and 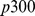 on 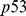


The steady state solutions in deterministic case can be obtained by putting the conditions 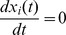, 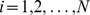, where 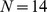, to the set of differential equations (3)–(16) and solving for various variables 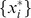. Following this procedure we first solve for 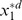 (steady state solution of p53) as a function of 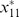 (steady state solution of HDAC1). The result is given by,
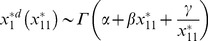
(31)where, 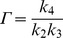, 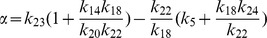, 
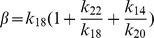
 and 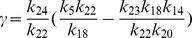 are constants. The equation (31) shows that the increase in 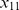 leads to increase in 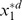 and second term in the equation is the main contributer. The reason being as 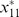 increases the third term 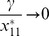 and the first term is a constant. Further, increase in 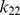 (degradation rate of HDAC1) and 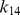 (degradation rate of p300) contribute increase in 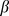, and therefore increases the steady state level of 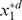. From the expression of 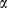, one can see that if 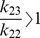 (p300 synthesis rate is larger than HDAC1 degradation rate), 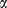 will contribute positive to 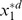, otherwise it will give negative contribution.

Proceeding in the same way, the steady state solution of 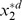 (Mdm2) can be obtained as a function of 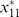. The result is given by,


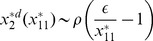
(32)

where, 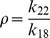 and 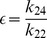
are constants. It can also be seen from the equation (32) that 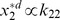 (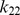 is degradation rate of HDAC1). Further for positive 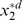, we have the condition 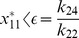 which means that the creation rate of HDAC1 (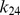) should be larger than degradation rate of HDAC1 (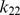) provided the condition. This behaviours can be seen in Fig. 8.

Next we solve for steady state solution 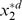 of 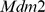 as a function of 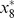 (steady state solution of p300) to study the impact of p300 on Mdm2. The result is given by,
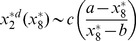
(33)where, 
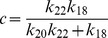
, 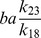 and 
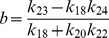
 are constants. From equation (33) for positive 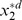 one can either 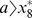 and 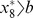 or 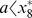 and 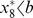. Moreover 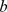 to be positive the condition 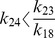 should be satisfied.

Now we solve steady state solution of 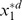 as a function of 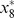 to understand the impact of p300 on p53. The result is given by,
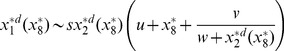
(34)where, 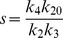, 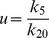, 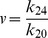 and 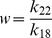 are constants. 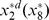 is given by the equation (33). The equation (34) indicates that 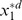 is increased by increase in 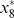 but decrease in 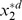. Further if 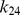, the sysnthesis rate of HDAC1 is increased then 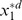 will also be increased. It can also be seen from 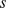 and (34) that 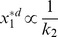 (synthesis rate of Mdm2).

### The role of noise and stabilization on 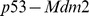 regulation

**Figure 14:**
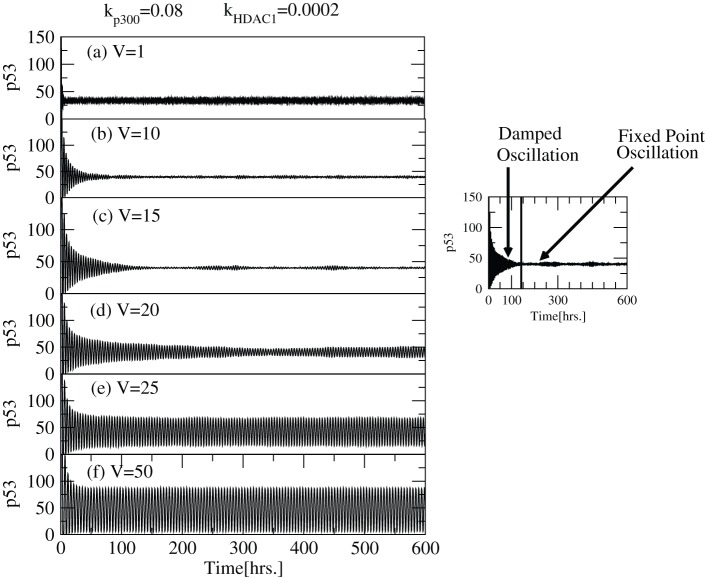
Noise contribution on 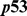 dynamics in stochastic system. The variation of 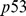 as a function of time in hours in stochastic system for different values of system size, 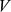 = 1, 10, 15, 20, 25, 50 (at constant values of 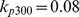 and 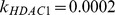).

Now we present the role of noise on 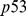 and 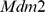 dynamics. This is done by solving the CLE equations (16)-(29) numerically. The results for different system size parameter, 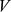 (1-50) at constant values of 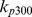 and 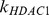, are shown in Fig. 14 (a)–(f). It has been observed that for 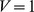, no oscillation in 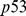 is seen. However, as 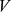 increases the oscillation starts emerging and when 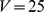 and 50 sustained oscillations are observed with increasing 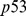 level. After 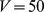 i.e. for 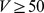, the 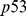 level remains constant i.e. it exhibits sustained oscillatory behaviour. The 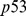 dynamics is noise induced stochastic process and the strength of noise decreases as 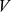 increases. The same behaviour is also seen in 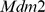 dynamics keeping all conditions the same (Fig. 14 (a)–(f)).

**Figure 15:**
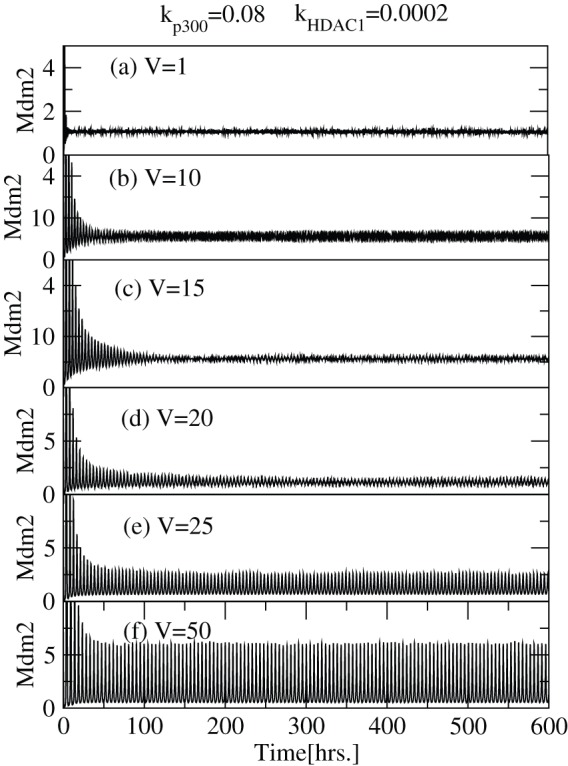
Noise contribution on 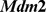 dynamics in stochastic system. The variation of 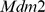 as a function of time in hours in stochastic system for different values of system size, 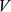 = 1, 10, 15, 20, 25, 50 (at constant values of 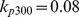 and 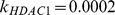).

Now we present the impact of 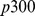 on 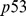 and 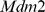 in stochastic system by simulating 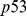 and 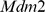 levels as a function of 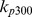 for different 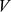 (Fig. 15). The result for 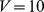 shows similar pattern as we found in the deterministic case, but the two conditions of stabilization and activation is achieved earlier with respect to 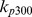 in stochastic case than that of the deterministic case as shown in the insets of the Fig. 15. Further, as one increases 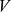, the values 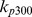 for getting the two conditions of stabilization and activation are increased.

**Figure 16:**
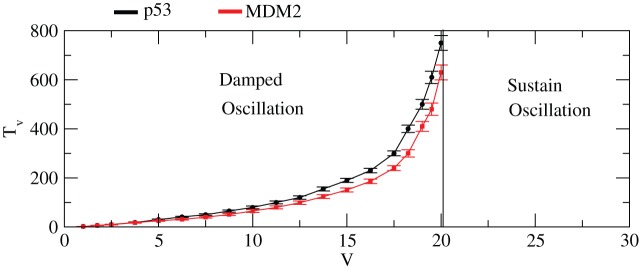
Phase diagram on 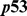 and 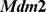 dynamics in stochastic system. Phase diagram indicating damped and sustained oscillation regimes induced by system size, 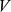.

The dynamics of 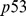 concentration remains constant with small fluctuations around the constant values of 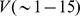 even though there is a small damping behavior at initial few hours. We then define 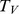 as the critical time below which the dynamics either shows damped or fixed point (stabilized) oscillations. The plot 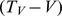 in Fig. 16 shows the damped, stabilized and oscillatory regimes. To generate this plot we took 50 simulations for a certain fixed set of parameters and points in the curves show average values with error bars which are correct up to of the order of 5-10 percent in our calculation as shown in Fig. 16. The plots show how system size, which can be taken as noise parameter (as V increases noise strength decreases and vice versa), drives the system at different states, namely, damped, stabilized (no oscillation) and sustain oscillation regimes.

We also study the impact of exposure time (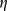) on 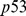 activation and stabilization for different values of 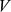 keeping the value of 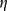 constant. We can see from the two left panels with insets in Fig. 15 that as 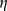 increases the conditions of stabilization and activation are obtained faster.

**Figure 17:**
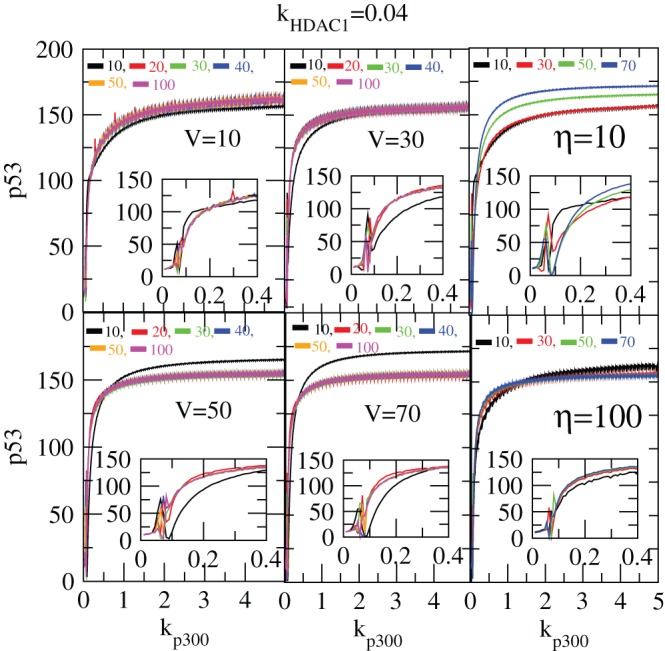
Stabilization of 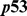 in stochastic system. (a) Plots of 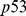 concentration levels as a function of 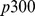 for different values of system size, V = 10, 30, 50 and 70 and for different values of 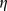 = [10–100] as shown in the four left panels. The insets show the enlarged portions of the activated regimes in each case. (b) Plots of 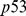 level versus 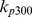 for different V = 10, 30, 50, 70 and for two different values of 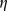 = 10 and 100 respectively as shown in two right hand panels.

**Figure 18:**
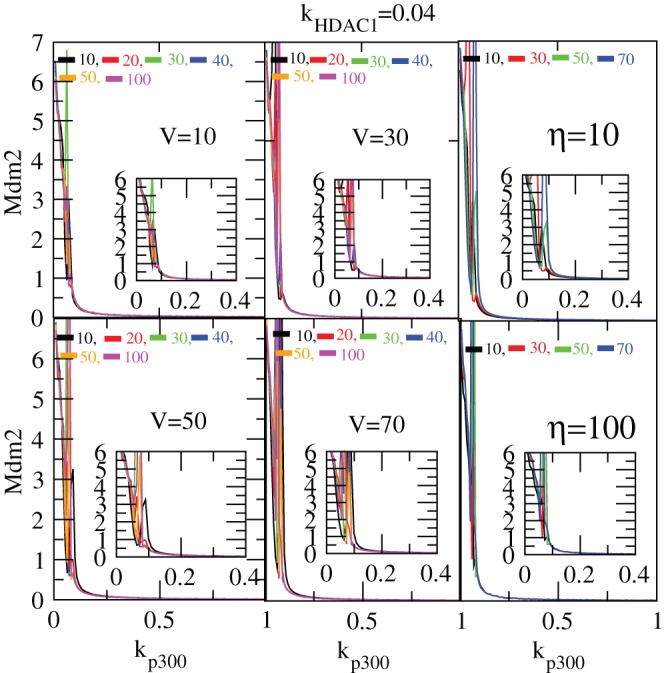
Stabilization of 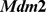 in stochastic system. (a) Plots of 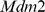 concentration levels as a function of 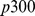 for different values of system size, V = 10, 30, 50 and 70 and for different values of 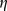 = [10–100] as shown in the four left panels. The insets show the enlarged portions of the activated regimes in each case. (b) Plots of 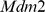 level versus 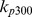 for different V = 10, 30, 50, 70 and for two different values of 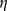 = 10 and 100 respectively as shown in two right hand panels.

The results showing the impact of 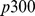 on 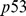 in stochastic system for different 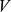
s and 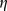
s are presented in Fig. 17. We also get the similar behaviour in the case as obtained in the case of 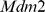 as shown in Fig. 18.

### Stochastic steady state solutions: the noise effect

The steady state solutions of CLE can also be obtained as we did in deterministic case from the equations (17)–(30). We first impose steady state condition to the set of CLEs i.e. 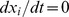 and got a set of steady state equations which are very difficult to solve. However, the steady state solutions can be obtained if we neglect negligible terms which have 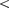 and 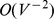 and rearrange the terms to solve the equations. Then one can easily solve simplified steady state equations. Proceeding in this way, the stochastic steady state solution of 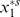 as a function of 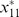 is obtained and given by,
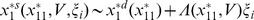
(35)where, 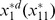 is given by equation (31) and we have taken the noise parameters 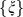 associated with each noise term are taken to be the same as 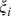. The noise term 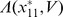 is given by,


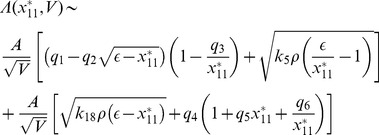
(36)

where, 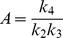, 
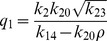
, 
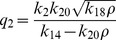
, 
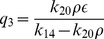
, 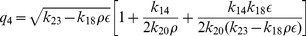, 
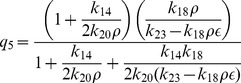
, and 
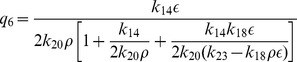
 are constants. It can be seen from equation (36) that the terms apart from first term and last terms in the last paranthesis will contribute to 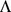 only when 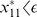. Hence for 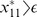, the equation (36) will have the following expression,
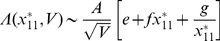
(37)where, 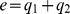, 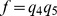 and 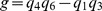. It can also be seen from 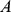 and equation (36)-(37)that 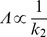.

Next we calculated the steady state solution of 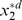 as a function of 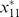. The result can be expressed along with the deterministic result as shown in equation (32) with noise term. It is given by,
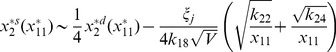
(38)where, 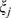 is the random noise parameter which we have taken same for all terms involved in the derivation. The noise contribution in this case is negative to the deterministic result which reduces steady state level of 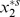 as the strength of noise increases. Further the increase in degradation and synthesis rate of HDAC1 (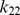) lead to increase in noise contribution which in turn decreases 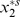.

Similarly, the stochastic steady state solutions of 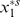 and 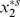 as a function of  along with their respective deterministic solutions given by equations (33) and (34) can also be calculated. The results are given by,
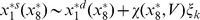
(39)and
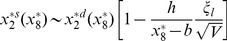
(40)where, 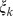 and 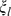 are random noise parameters for equations (39) and (40) respectively. The function 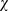 is given by



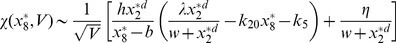


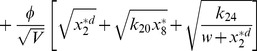
(41)where, 
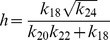
, 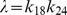, 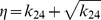 and 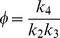 are constants. The noise function 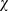 is mainly contributed from first, 5th and 6th terms in equation (41) and 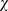 is positive contributor to the deterministic part. From these main contributing terms, the synthesis rate of HDAC1, 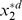 and 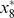 and their variation give significant contributions to the noise terms in equations (39) and (40). However noise contribution in equation (40) is negative contributor to the deterministic part.

## Conclusion

The interaction of 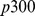 with 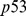 allows 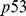 to be acetylated which prohibits it from decaying and allows it to participate in other reactions. This excess in 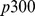 level eventually leads to increase in capped 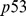 whose population cannot be controlled and subjects the cell to stress condition. If the excess in 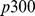 level is strong enough it may lead to cell death due to uncontrolled 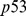, similar to cancer. We observe this phenomena in our simulation results in qualitative sense via three different stages/conditions, namely, first stabilization or normal condition where impact of 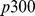 is negligible, second activation of 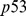 due to significant interaction between 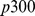 and 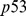, and third uncontrolled growth of capped 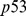 due to interaction with excess 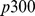 leading to second stabilization level which may represent cell death condition. The same behaviour is seen in 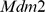 simulation results. The three conditions of stabilization and activation are obtained but the second stabilization level is obtained at lower level as compared to first stabilization level. This may be due to the fact that the increase of capped 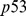 cannot activate 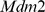 as is done normally, and goes to lower minimum level.

The interaction of 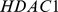 with 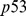 will cause deacetylation of capped 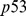 which leads 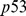 to participate in other reactions and able to decay. This may help the already stressed cell to bring back to its normal condition. However excess of 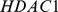 will cause excess deacetylation of 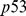 and will allow the cell to come back far beyond to its normal condition leading to stress. Our results supports these findings.

Noise has interesting but contrasting roles in stochastic system depending upon its strength. If its strength is strong then it has destructive impact on the signal processing in and outside the system etc. However if its strength is weak then it exhibit constructive role, for example weak signal detection, amplification and processing the signal etc. In our study, we found that if the system size is very small where the noise strength is very strong with respect to system size, the associated noise destroy the signal in the system which is in agreement with the theoretical claim. But if the system size is increased in our study where noise strength is comparatively weaker, the signal is resumed in normal with noise induced dynamics in each variable. Moreover, in stochastic system, the 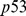/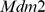 is activated by small concentration level of 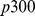/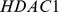 as compared to those in deterministic case and reach stabilization much much faster as compared to deterministic system. Further increase in system size reduces the noise fluctuation in the dynamics of each variable and when 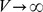, the noise strength is negligible and the system goes to classical deterministic system.

In the present study we determine only the impact of 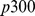 and 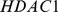 on 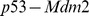 regulatory network. For developing any realistic model one needs to incorporate other proteins which influence 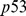 protein simultaneously and then study the impact collectively. Our study is just one step forward towards understanding p53 regulatory network.
